# Silencing Herpes Simplex Virus Type 1 Capsid Protein Encoding Genes by siRNA: A Promising Antiviral Therapeutic Approach

**DOI:** 10.1371/journal.pone.0096623

**Published:** 2014-05-02

**Authors:** Fujun Jin, Shen Li, Kai Zheng, Cuiqin Zhuo, Kaiqi Ma, Maoyun Chen, Qiaoli Wang, Peizhuo Zhang, Jianglin Fan, Zhe Ren, Yifei Wang

**Affiliations:** 1 Guangzhou Jinan Biomedicine Research and Development Center, National Engineering Research Center of Genetic Medicine, Jinan University, Guangzhou, Guangdong, China; 2 College of Pharmacy, Jinan University, Guangzhou, Guangdong, China; 3 Department of Molecular Pathology, Interdisciplinary Graduate School of Medicine and Engineering, University of Yamanashi, Yamanashi, Japan; 4 Shanghai GenePharma Co., Ltd, Shanghai, China; Fudan University, China

## Abstract

Herpes simplex virus type 1 (HSV-1), a member of the *herpesviridae*, causes a variety of human viral diseases globally. Although a series of antiviral drugs are available for the treatment of infection and suppression of dissemination, HSV-1 remains highly prevalent worldwide. Therefore, the development of novel antiviral agents with different mechanisms of action is a matter of extreme urgency. During the proliferation of HSV-1, capsid assembly is essential for viral growth, and it is highly conserved in all HSV-1 strains. In this study, small interfering RNAs (siRNAs) against the HSV-1 capsid protein were screened to explore the influence of silencing capsid expression on the replication of HSV-1. We designed and chemically synthesized siRNAs for the capsid gene and assessed their inhibitory effects on the expression of target mRNA and the total intracellular viral genome loads by quantitative real-time PCR, as well as on the replication of HSV-1 via plaque reduction assays and electron microscopy. Our results showed that siRNA was an effective approach to inhibit the expression of capsid protein encoding genes including *UL18*, *UL19*, *UL26*, *UL26.5*, *UL35* and *UL38 in vitro*. Interference of capsid proteins VP23 (*UL18*) and VP5 (*UL19*) individually or jointly greatly affected the replication of clinically isolated acyclovir-resistant HSV-1 as well as HSV-1/F and HSV-2/333. Plaque numbers and intracellular virions were significantly reduced by simultaneous knockdown of *UL18* and *UL19*. The total intracellular viral genome loads were also significantly decreased in the *UL18* and *UL19* knockdown groups compared with the viral control. In conclusion, interfering with *UL18* and *UL19* gene expression could inhibit HSV-1 replication efficiently in vitro. Our research offers new targets for an RNA interference-based therapeutic strategy against HSV-1.

## Introduction

Herpes simplex virus type 1 (HSV-1), a member of the *herpesviridae* family, is a linear double-stranded DNA virus that only infects humans naturally, with no known animal host [1]. HSV-1 primarily affects the mucocutaneous surfaces and leads to persistent lesions. It can also infect the central nervous system or visceral organs and lead to disseminated infections, such as hepatitis, esophagitis, pneumonia and meningoencephalitis. Recently, an increasing proportion of genital infections were reported also to be caused by HSV-1 in some developed countries [2]–[4]. In the United States, approximately 50–70% of healthy adults and 20–40% of healthy children are infected with HSV-1 [5]. After primary infection, HSV-1 establishes long-term latency in the ganglia of sensory nerves. It can reactivate when the immune system is deficient or stimulated by various factors. These reactivations may be accompanied by symptoms or clinically silent [6]. HSV-1 infections can cause considerably severe symptoms in immunocompromised patients. In patients with human immunodeficiency virus type 1 (HIV-1) infection or in recipients of organ transplants, herpetic lesions can be extensive, persistent and more easily disseminated with increased recurrences [7]. Despite intensive efforts, current treatment strategies remain inadequate for controlling HSV-1 infections. Since its first application in the 1980s, acyclovir (ACV) and other derivatives have been approved worldwide for the treatment and suppression of herpetic infections [5], [8]–[9]. Problems related with toxicity and drug resistance have long been reported, and the emergence of resistance to ACV has created an obstacle for the treatment of HSV-1 [10]–[11]. Therefore, the development of new anti-HSV agents with different mechanisms of action is a matter of great urgency.

The viral capsid is an essential structural component of HSV-1 virus particles. It plays a critical role in virion replication, assembly, maturation and infection [12]–[13]. Assembly of herpesvirus capsids takes place in the nucleus. After the procapsid is formed, the viral genome is packaged into it. There are three distinct types of capsids contained in host cells, type A, type B and type C. Among these, only type C capsids contain the viral DNA [14]. The mature HSV-1 capsid is shaped like an icosahedral shell, with a diameter of 125 nm and a thickness of 15 nm. It consists of 162 capsomeres, including 150 hexons, 11 pentons and one portal that consists of a dodecamer of pUL6 [15]. The mature capsid shell is composed of four predominant protein components, a major capsid protein (VP5) and three less abundant proteins (VP19C, VP23 and VP26). VP19C and VP23 together make up a triplex, which acts as a scaffold during the formation of the HSV-1 capsid [16]–[18]. In addition to the shell proteins mentioned above, HSV-1 B capsids contain a large amount of the scaffolding protein (product of the *UL26.5* gene) and smaller amounts of the two products encoded by *UL26*, VP24 and VP21 [13], [18]. The crucial roles of capsids in HSV-1 maturation implicates them as hopeful therapeutic targets, and studies are underway to identify effective inhibitors [19].

RNA interference (RNAi) is a gene-silencing mechanism, which is induced by small double-stranded RNA [20]. It was initially observed in *Caenorhabditis elegans* [21] and then later found to exist in plants [22] and mammalian cells as well [23]. Small interfering RNAs (siRNAs), double-stranded RNAs of 21–25 nucleotides in length generated by the ribonuclease III enzyme Dicer, act as functional intermediates in RNAi to induce target mRNA cleavage by the RNA-induced silencing complex. This powerful technology has been widely used to manipulate gene expression, identify gene functions on a whole-genome scale and develop antiviral strategies for prevention and treatment of human viral diseases [23]–[25]. To date, RNAi technology has been developed against several human pathogens, including HIV-1, hepatitis B virus, hepatitis C virus, influenza A virus, poliovirus and Dengue virus [26].

In the present study, we applied siRNAs targeting the HSV-1 capsid to research the effects of knocking down these genes on HSV-1 replication and infection. The results showed that knockdown of major capsid proteins VP5 and VP23 encoded by *UL19* and *UL18*, respectively, could greatly affect HSV-1 proliferation. Our findings suggest that RNAi targeting VP5 and VP23 is an effective therapeutic alternative strategy against HSV-1 infection.

## Materials and Methods

### Cells and Viruses

African green monkey kidney cells (Vero, ATCC CCL81), obtained from the Wuhan Institute of Virology, Chinese Academy of Sciences, were cultured in Dulbecco’s modified Eagle medium (DMEM) (Gibco) supplemented with 10% heat-inactivated fetal bovine serum (FBS) (Gibco). The constituents of the maintenance medium were DMEM supplemented with 2% FBS. The cells were cultured at 37°C in a humidified atmosphere with 5% CO_2_. HSV-1/F (ATCC VR-733) was provided by Hong Kong University. HSV-2/333 was obtained from the Wuhan Institute of Virology, Chinese Academy of Sciences. HSV-1/Blue, a TK mutant derived from HSV-1 (KOS) and two ACV-resistant clinical HSV-1 strains HSV-1/106 and HSV-1/153 [27] were a kind gift from Tao Peng, State Key Laboratory of Respiratory Disease, Guangzhou Institutes of Biomedicine and Health, Chinese Academy of Sciences. All viruses were propagated in Vero cells and stored at −80°C until use. Virus titers were determined by a 50% tissue culture infectious dose (TCID_50_) assay on Vero cells as reported previously [28]. The TCID_50 _ml^−1^ measurements were used with a subsequent conversion to plaque-forming units (PFU) ml^−1^ as described by Dougherty [29].

### siRNA Design and Transfection

The siRNAs targeting genes of the HSV-1 capsid proteins, including *UL18*, *UL19*, *UL26*, *UL26.5*, *UL35* and *UL38*, were designed using online siRNA programs: siRNA Selection Program (http://sirna.wi.mit.edu/home.php), siDirect version 2.0 (http://sidirect2.rnai.jp/) and Deqor v3 (http://deqor.mpi-cbg.de/deqor_new/input.html). The siRNAs used for downregulation of the capsid genes as well as the negative control siRNA (Table 1), which does not target any sequence present in HSV or the human genome, were obtained from Shanghai GenePharma Co., Ltd. Transient transfection of siRNA was performed with the siRNA-Mate transfection regent (Genepharma) according to the manufacturer’s instruction.

**Table 1:** siRNAs targeting HSV-1 capsid proteins.

siRNA	Sequence (sense, antisense)	Target gene
siUL18-1	GCACCGUUAACCUUCGCAATT UUGCGAAGGUUAACGGUGCTT	*UL18*(VP23)
siUL18-2	GUCCUUAACAUGGUUUACUTT AGUAAACCAUGUUAAGGACTT	*UL18*(VP23)
siUL18-3	CCAUCAUCCUUACGCUAAUTT AUUAGCGUAAGGAUGAUGGTT	*UL18*(VP23)
siUL18-4	CCCGUUAUACGCUAUCCCUAA AGGGAUAGCGUAUAACGGGGG	*UL18*(VP23)
siUL19-1	CCAGCGACGUACAGUUUAATT UUAAACUGUACGUCGCUGGCG	*UL19*(VP5)
siUL19-2	CUUUGUUGUUGCCGAUGCATT UGCAUCGGCAACAACAAAGTT	*UL19*(VP5)
siUL19-3	CGACCGACGUCAACUACUUTT AAGUAGUUGACGUCGGUCGTT	*UL19*(VP5)
siUL19-4	CCAGCGACGUACAGUUUAATT UUAAACUGUACGUCGCUGGTT	*UL19*(VP5)
siUL26-1	CCGUUAACAACAUGAUGCUTT AGCAUCAUGUUGUUAACGGCG	*UL26*(VP21/24)
siUL26-2	CCGAUUUGUUCGUCUCUCATT UGAGAGACGAACAAAUCGGCG	*UL26*(VP21/24)
siUL26-3	CUGUUGUACCUGAUCACCAAC UGGUGAUCAGGUACAACAGGC	*UL26*(VP21/24)
siUL26-4	CCGUUAACAACAUGAUGCUGC AGCAUCAUGUUGUUAACGGCG	*UL26*(VP21/24)
siUL26.5	CCGAUUUGUUCGUCUCUCAUU UUGGCUAAACAAGCAGAGAGU	*UL26.5*(ICP35)
siUL35-1	CACGCAAACAACACGUUUATT UAAACGUGUUGUUUGCGUGGG	*UL35*(VP26)
siUL35-2	GCCACCAAUAACUCUCAGUTT ACUGAGAGUUAUUGGUGGCCA	*UL35*(VP26)
siUL35-3	CUCUCAGUUUAUCAUGGAUTT AUCCAUGAUAAACUGAGAGTT	*UL35*(VP26)
siUL35-4	GUUUGUCGUUCGAGAACCUTT AGGUUCUCGAACGACAAACGG	*UL35*(VP26)
siUL38-1	GGCCUAGUGUCGUUUAACUTT AGUUAAACGACACUAGGCCCG	*UL38*(VP19C)
siUL38-2	GGAUCACCAACACGAUUCATT UGAAUCGUGUUGGUGAUCCGG	*UL38*(VP19C)
siUL38-3	GCGUUUCUGUACCUGGUAUTT AUACCAGGUACAGAAACGCCG	*UL38*(VP19C)
siUL38-4	GUUGUGUGUACGUGAUCAATT UUGAUCACGUACACACAACAC	*UL38*(VP19C)
siN.C	UUCUCCGAACGUGUCACGUTT AGGUGACACGUUCGGAGAATT	Negative control

### RNA Isolation, Reverse Transcription and Quantitative Real-time PCR (qPCR)

Vero cells were grown in 6-well plates to 70–80% confluency and then transfected with various siRNAs (200 pmol/well) using siRNA-Mate. After 4–6 h, cells were infected with HSV-1 at the multiplicity of infection (MOI) of 5. After 24 h, total RNA from infected cells was extracted using Trizol (Invitrogen). RNA concentrations were measured using a spectrophotometer (Thermo) at wavelengths of 260 and 280 nm. Extracted RNA (500 ng) was reverse transcribed into cDNA using a PrimeScript RT reagent kit (Takara). The qPCR assays were conducted using SsoFast EvaGreen Supermix (Bio-Rad) according to the manufacturer’s instructions. Primer pairs used were specific for *UL18* (F: 5′-TGG CGG ACA TTA AGG ACA TTG -3′ and R: 5′-TGG CCG TCA ACT CGC AGA-3′), *UL19* (F: 5′-GAC CGA CGG GTG CGT TAT T-3′ and R: 5′-GAA GGA GTC GCC ATT TAG CC-3′), *UL26* (F: 5′-GCC TTC TTC GCC TTT CGC-3′ and R: 5′-CGC TCG TGC CCT TCT TCT T-3′), *UL26.5* (F: 5′- CCT ATG GGC CTC ACG GCG C -3′ and R: 5′- AAC GCG GCT ATC TGC GCC TC -3′), *UL35* (F: 5′-CGG GTG TTC GTC GTC TTC GG-3′ and R: 5′-CCC GTC TTC ATG TAT GGC GAG T-3′), *UL38* (F: 5′-CGC GGC GTT TCT GTA CCT G-3′ and R: 5′-TGC CGT GAA TCG TGT TGG TG-3′) and *GAPDH* (F: 5′-CCC ACT CCT CCA CCT TTG AC-3′ and R: 5′-TCT TCC TCT TGT GCT CTT GC-3′). The relative expression of each gene was normalized to the housekeeping gene *GAPDH* and calculated as reported previously [30].

### Cytotoxicity Assays

Cytotoxicity of the siRNA-Mate transfection reagent and siRNAs on Vero cells was determined using 2-(2,5-dimethyl-2-thiazolyl)-2,5-diphenyl-2H-tetrazolium bromide (MTT) assays. Mixes of different siRNA-Mate:siRNA ratios were transfected into Vero cells cultured in 96-well plates according to the manufacturer’s instruction, with each transfection ratio was tested in triplicate. At 24 h post-transfection, 10 µl MTT stock solution (5 mg/mL) was added to each well, and the plate was incubated for 4 h in the dark. After the MTT solution was discarded, 100 µl dimethyl sulfoxide (DMSO) was added to each well, and plates were gently shaken for 15 min at room temperature. The optical density (OD) of each well was measured with an enzyme immunoassay (EIA) reader (Bio-Rad) at 570 and 630 nm. The cell viability of each group was determined by comparison with the cell control.

### Fluorescence Microscopy

Vero cells were seeded in 24-well cell culture plates and grown to 70% confluency for siRNA transfection. FAM-labeled siRNA (siN.C-FAM F:5′-UUC UCC GAA CGU GUC ACG UTT-3′ R:5′-AGG UGA CAC GUU CGG AGA ATT-3′) was transfected into Vero cells at the indicated concentrations. On various days after transfection, images were acquired using an OLYMPUS IX71 fluorescence microscope (OLYMPUS).

### Plaque Formation Assay

Vero cells were grown in 24-well plates to 70% confluency and then transfected with various siRNAs (40 pmol) using siRNA-Mate. After 4 h, cells were infected with 30–40 PFUs of different HSV-1 strains. The virus suspension was discarded 2 h later, and cells were washed with PBS and then overlaid with 1 ml of a 1∶1 mixture of sodium carboxymethylcellulose (NaCMC):DMEM lacking serum. At 72 h post-infection, plates were fixed with 10% paraformaldehyde for 15 min and stained with 1% crystal violet for 20 min. Plaques were enumerated in each well, and images of the monolayers were acquired. Overlapping plaques and plaques at the edges of a well were counted as a single plaque. Plaques were counted, and the plaque reduction ratio was calculated using the following formula: plaque reduction ratio  =  (plaques of virus control group-plaques of siRNA group)/(plaques of virus control group) ×100%.

### Electron Microscopy

Vero cells were grown in 100-mm cell culture plates to 70% confluency and then transfected with siRNA (1500 pmol/plate) using siRNA-Mate. After 6 h, cells were infected with HSV-1 at the MOI of 20 to 30 for 2 h for viral absorption. After absorption of the virus, the medium was changed to growth medium, and cells were harvested at 24 h post-infection. The collected cells were first fixed in 3.0% glutaraldehyde (pH 7.2) for 1.5 h, post-fixed in 1% osmium tetroxide for 1 h, followed by dehydration and embedding in Spurr (Sigma). Ultrathin sections were cut and stained with aqueous uranyl acetate and lead citrate and observed with a transmission electron microscope JEM1400 (JEOL).

### Measurement of Intracellular Viral Genome Copy Number

Vero cells were grown in 6-well plates to 70–80% confluency and then transfected with various siRNAs (200 pmol/well) using siRNA-Mate. After 4–6 h, cells were infected with HSV-1 at the indicated multiplicity of infection (MOI). At different hours post-infection the cells were washed three times with PBS and then digested and recovered by centrifugation. The internalized viral DNA was isolated using a GeneJET Viral DNA and RNA Purification Kit (Thermo). The intracellular HSV-1 genome copy numbers were assessed by detecting the viral *UL47* gene using real-time DNA PCR and were expressed relative to the virus control groups. Primer pairs used to detect *UL47* were as follows: F: 5′-GAC GTA CGC GAT GAG ATC AA -3′ and R: 5′-GTT ACC GGA TTA CGG GGA CT-3′.

### Statistical Analysis

Data was analyzed using GraphPad Prism 5 (GraphPad Software, La Jolla, CA, http://www.graphpad.com). Results were calculated as the mean ± SEM, and statistical significance were determined by the Student’s t test. *P* values of less than 0.05 were considered statistically significant.

## Results

### Optimal Transfection Conditions

**Figure 1:**
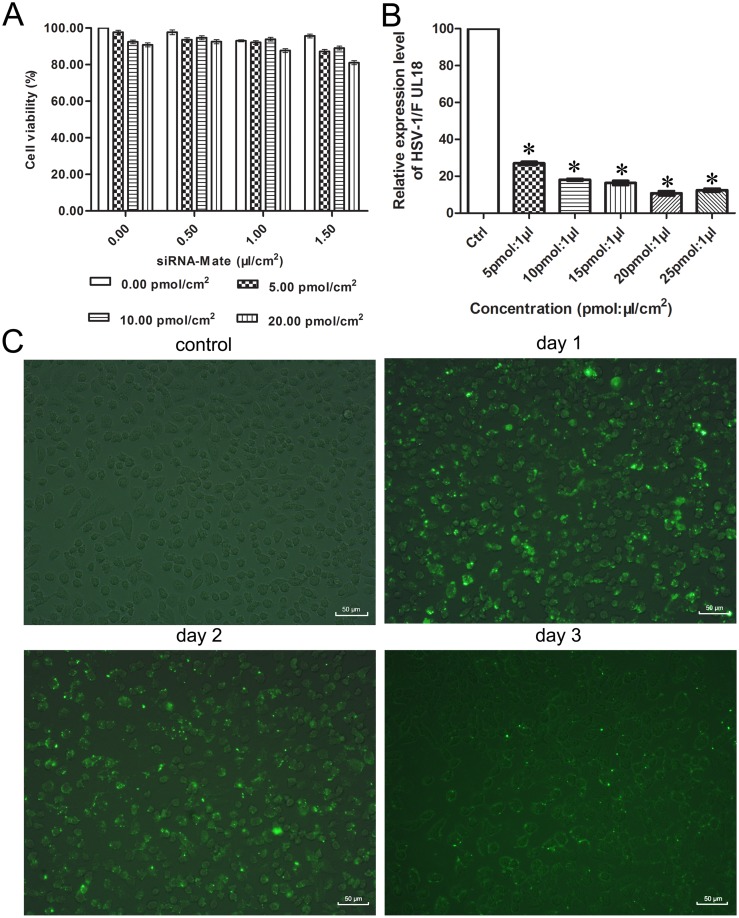
Optimal transfection conditions. (A) Cell viability with siRNA and transfection reagent siRNA-Mate at different concentrations was assessed with the MTT assay. (B) qPCR was conducted to analyze the knockdown efficiency of transfecting different ratios of siRNA:siRNA-Mate/cm^2^. Each sample was analyzed in triplicate, and data are expressed as the mean ± SEM. *P<0.05 vs. virus control group. (C) FAM-tagged siRNA was transfected into Vero cells with a transfection ratio of 20 pmol siRNA:1 µl siRNA-Mate/cm^2^. The transfection efficiency and durability of the siRNA were detected by fluorescence microscopy on days 1, 2 and 3 after transfection.

Cytotoxicity levels of the siRNAs and transfection regents, as measured by the MTT assay, were very low even in high concentrations (Figure 1A). The cell viability of the 20 pmol siRNA: 1.5 µl siRNA-Mate/cm^2^ groups reached up to 81%. To screen for the most efficient knockdown ratios of siRNA:siRNA-Mate, an effective siRNA targeting the capsid protein VP23 encoding gene *UL18*, siUL18-1 (data from pre-experiment), was chosen as a representative of siRNAs for further experiments. The interference efficiency for each group approached 70%. The highest interference efficiency was obtained using 20 pmol of siRNA with 1 µl of siRNA-Mate/cm^2^ (Figure 1B), and this transfection ratio was used in further experiments. The transfection efficiency and durability of siRNAs in cells were determined by fluorescence microscopy. As shown in Figure 1C, the transfection efficiency reached up to 95% in this condition on the first day. Two days after transfection, the percentage of positive cells was maintained at 70%, but it decreased to 20% three days later.

### Effects of Different siRNAs Targeting Capsid-related Genes on HSV-1 Proliferation

**Figure 2:**
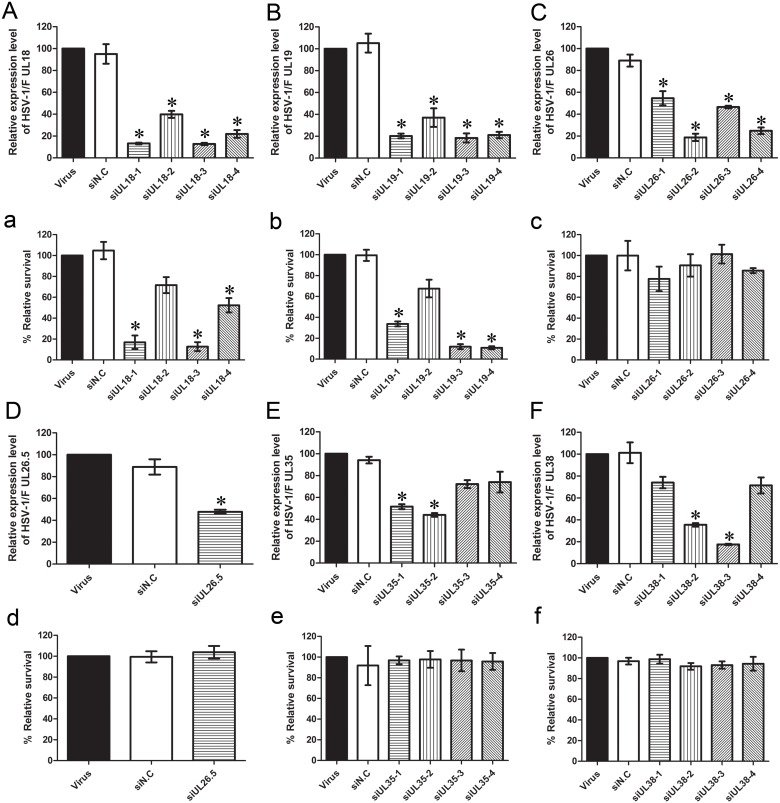
Effect of siRNA on HSV-1 plaque formation and relative expression of capsid-related genes in HSV-1-infected Vero cells. (A–F) Effects of siRNA on relative mRNA expression levels of capsid genes (*UL18*, *UL19*, *UL26*, *UL26.5*, *UL35* and *UL38*, respectively) compared with the virus group were analyzed by qPCR. (a–f) Effects of siRNA against different capsid genes (*UL18*, *UL19*, *UL26*, *UL26.5*, *UL35* and *UL38*, respectively) on HSV-1 proliferation were evaluated by a plaque reduction assay. Vero cells were treated with different siRNAs. Cultures were infected with HSV-1, and plaques were counted 72 h later. The relative survival rate of each group was compared with the virus control set at 100%. Each sample was analyzed in triplicate, and data are expressed as the mean ± SEM. *P<0.05 vs. viral group.

In this experiment, 21 pairs of siRNAs targeting the HSV-1 capsid protein encoding gene were designed to evaluate the therapeutic efficiency of siRNA against HSV-1, including *UL18* (capsid triplex protein VP23), *UL19* (major capsid protein VP5), *UL26* (capsid maturation protease VP24/21), *UL26.5* (capsid scaffold protein VP22a), *UL35* (small capsid protein VP26) and *UL38* (capsid triplex protein VP19C). First, qPCR was performed to determine whether the siRNAs could efficiently silence the expression of these genes. As shown in Figure 2, *UL18*-specific siRNAs inhibited the expression of the *UL18* gene significantly compared with the virus control. Expression of the *UL18* gene dropped to 13.22% and 12.75% with the use of siUL18-1 and siUL18-3, respectively (Figure 2A). The equivalent amounts of UL18, siUL19-1, siUL19-3 and siUL19-4 reduced the expression of the *UL19* gene to 20.29%, 18.48% and 21.14%, respectively (Figure 2B). Expression of the *UL26* gene decreased to 18.79% with the use of siUL26-2 (Figure 2C), while that of the *UL26.5* gene was reduced to 47.88% by siUL26.5 (Figure 2D). siUL35-2 reduced the expression of the *UL35* gene to 44.05% (Figure 2E), and siUL38-3 reduced that of the *UL38* gene to 17.52% (Figure 2F). The negative control group showed no statistically significant difference compared with the virus-infected groups (*P*>0.05).

To evaluate the effect of siRNA on HSV-1 infection, the plaque reduction assay was performed, which is the gold standard phenotypic method for the evaluation of HSV susceptibility to antiviral drugs [31]. Vero cells were transfected with different siRNAs or negative control siN.C and then infected with 30–40 PFU of HSV-1 as described above. Compared with the virus control, siN.C had no effect upon viral plaque formation after infection. In contrast, the groups transfected with *UL18*- and *UL19*-specific siRNAs produced much fewer plaques. The inhibition rates of siUL18-1, siUL18-3, siUL19-1, siUL19-4 and siUL19-5 on HSV-1 plaque formation were 83.02%, 87.26% (Figure 2a), 76.30%, 88.03% and 89.13% (Figure 2b), respectively. However, compared with *UL18* and *UL19*, knockdown of *UL26* (Figure 2c), *UL26.5* (Figure 2d), *UL35* (Figure 2e) and *UL38* (Figure 2f) did not have large effects on the proliferation of HSV-1.

### VP23 and VP5 Knockdown Inhibits Proliferation of ACV-resistant HSV-1 Strains and HSV-2

**Figure 3:**
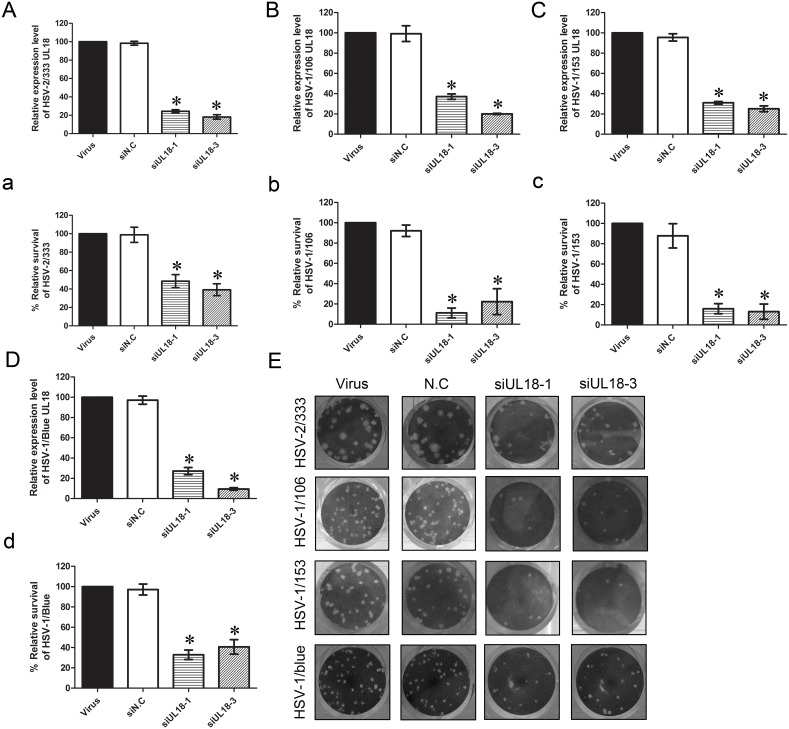
Effect of siUL18 on plaque formation and relative *UL18* gene expression of acyclovir-resistant HSV-1 strains and HSV-2 333. (A–D) Effects of siUL18 on relative mRNA expression of the *UL18* gene were analyzed by qPCR compared with the virus group (HSV-2/33, HSV-2/106, HSV-1/153 and HSV-1/Blue, respectively). (a–d) Effects of siUL18 on proliferation of acyclovir-resistant HSV-1 and HSV-2 333 were evaluated by a plaque reduction assay. Cells were treated with siUL18 and infected with each virus (HSV-2/33, HSV-2/106, HSV-1/153 and HSV-1/blue, respectively). Plaques were counted 72 h later. The relative survival rate of each group was compared with the virus control set at 100%. Each sample was analyzed in triplicate, and data are expressed as the mean ± SEM. *P<0.05 vs. viral group.

**Figure 4:**
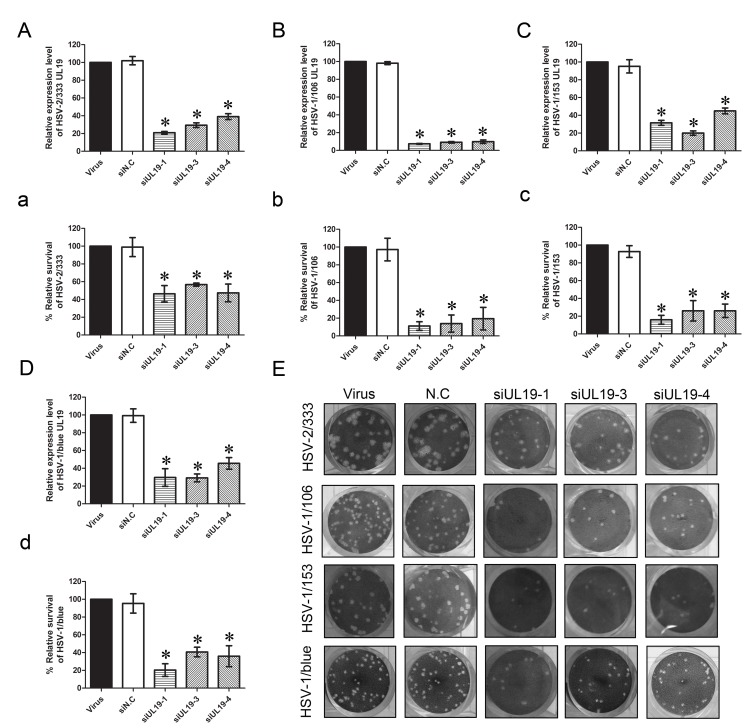
Effect of siUL19 on plaque formation and relative *UL19* gene expression of acyclovir-resistant HSV-1 strains and HSV-2 333. (A–D) Effects of siUL19 on relative mRNA expression level of the *UL19* gene were analyzed by qPCR compared with the viral group (HSV-2/33, HSV-2/106, HSV-1/153 and HSV-1/Blue, respectively). (a–d) Effects of siUL19 on proliferation of acyclovir-resistant HSV-1 and HSV-2 333 were evaluated by a plaque reduction assay. Cells were treated with siUL19 and infected with each virus (HSV-2/33, HSV-2/106, HSV-1/153 and HSV-1/blue, respectively). Plaques were counted 72 h later, and the relative survival rate of each group was compared with virus control set at 100%. Each sample was analyzed in triplicate, and data are expressed as the mean ± SEM. *P<0.05 vs. viral group.

In the previous experiments, we found that interference of *UL18* and *UL19* expression could significantly affect the proliferation of HSV-1 and that VP23 and VP5 capsid proteins play crucial roles in the process of capsid assembly and DNA packaging of HSV. Therefore, we speculated that *UL18* (VP23) and *UL19* (VP5) can be potential drug targets, but whether knockdown of these genes can also suppress the proliferation of clinically isolated ACV-resistant HSV-1 strains and HSV-2 strains has not been reported. Here, two clinically isolated ACV-resistant HSV-1 strains HSV-1/106 and HSV-1/153, as well as HSV-1/Blue, a TK mutant strain derived from HSV-1 (KOS) [27] and the HSV-2 333 strain were used. We chose the best siRNA from several groups, including siUL18-1, siUL18-3, siUL19-1, siUL19-3, siUL19-4 for subsequent experiments. As described above, qPCR were performed to analyze the interference efficiency of these siRNAs on clinically isolated ACV-resistant HSV-1 strains and HSV-2, and the plaque reduction assay was performed to evaluate the effect of *UL18* and *UL19* knockdown on proliferation of these viruses. The results showed that *UL18*- and *UL19*-specific siRNAs could also downregulate the mRNA expression of *UL18* and *UL19* of these viruses (Figures 3A–D and Figure 4A–D). As shown in Figure 3E and Figure 4E, the siRNAs inhibited the viral plaque formation significantly after infection. Compared with the virus control, Vero cells transfected with *UL18*- and *UL19*-specific siRNAs produced much fewer plaques. Specifically, for the HSV-2/333 strain, siUL18-1 and siUL18-3 reduced the *UL18* gene expression to 24.38% and 18.17% (Figure 3A), while siUL19-1, siUL19-3 and siUL19-4 reduced the *UL19* gene expression to 20.92%, 29.40% and 39.02% (Figure 4A), respectively. Meanwhile, the inhibition rates of HSV-2/333 plaque formation by siUL18-1 and siUL18-3 were 51.55% and 60.82% (Figure 3a) and that by siUL19-1, siUL19-3 and siUL19-4 were 53.61%, 43.30% and 52.58% (Figure 4a), respectively. For the ACV-resistant HSV-1/106 strain, siUL18-1 and siUL18-3 reduced the *UL18* gene expression to 37.11% and 20.05% (Figure 3B), while siUL19-1, siUL19-3 and siUL19-4 reduced the *UL19* gene expression to 7.40%, 9.10% and 9.83% (Figure 4B), respectively. The inhibition rates of siUL18-1 and siUL18-3 on HSV-1/106 plaque formation were 88.89% and 78.78% (Figure 3b), and those of siUL19-1, siUL19-3 and siUL19-4 on HSV-1/106 plaque formation were 88.99%, 86.11% and 80.56% (Figure 4b), respectively. For the ACV-resistant HSV-1/153 strain, siUL18-1 and siUL18-3 reduced the expression of *UL18* gene of HSV-1/153 to 31.00% and 25.12% (Figure 3C), while siUL19-1, siUL19-3 and siUL19-4 reduced the *UL19* gene expression to levels of 31.52%, 20.00% and 44.94% (Figure 4C), respectively. The inhibition rates on HSV-1/153 plaque formation by siUL18-1 and siUL18-3 were 84.06% and 86.96% (Figure 3c), and those by siUL19-1, siUL19-3 and siUL19-4 were 84.06%, 73.91% and 73.91% (Figure 4c), respectively. For the TK mutant HSV-1/blue strain, siUL18-1 and siUL18-3 reduced the *UL18* gene expression to 27.17% and 9.47% (Figure 3D), while siUL19-1, siUL19-3 and siUL19-4 reduced the *UL19* gene expression to levels of 29.61%, 29.17% and 45.52% (Figure 4D), respectively. The inhibition rates on HSV-1/blue plaque formation by siUL18-1 and siUL18-3 were 67.19% and 59.37% (Figure 3d), and those by siUL19-1, siUL19-3 and siUL19-4 were 79.69%, 59.37% and 64.06% (Figure 4d), respectively.

### Interference with siUL18 and siUL19 Jointly Exhibits a more Potent Inhibitory Effects on Different HSV Viruses

**Figure 5:**
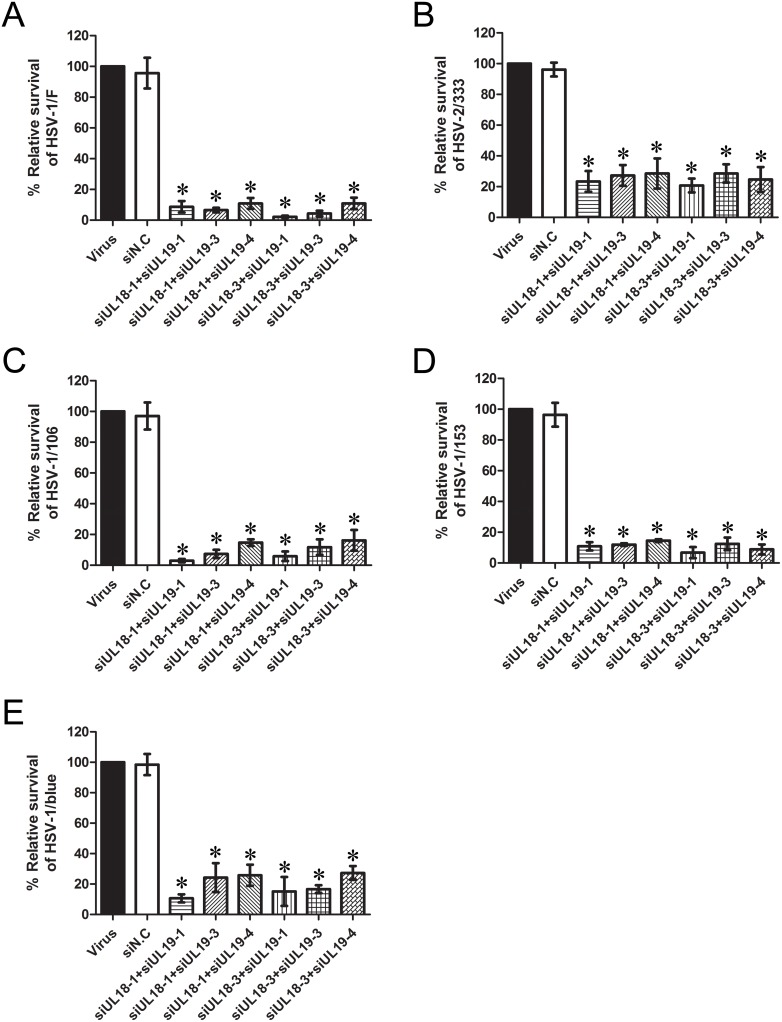
Joint inhibitory effects of siUL18 and siUL19 on different HSV strains. Vero cells were co-transfected with siUL18 and siUL19 each at a concentration of 10 pmol with 1 µl siRNA-Mate/cm^2^. Inhibitory effects were evaluated by a plaque reduction assay. Cell cultures were infected with (A) HSV-1/F, (B) HSV-2/33, (C) HSV-2/106, (D) HSV-1/153 or (E) HSV-1/blue, and plaques were counted 72 h later. The relative survival rate of each group was compared with the virus control set at 100%. Each sample was analyzed in triplicate, and data are expressed as the mean ± SEM. *P<0.05 vs. viral group.

Through the above experiments, we found obvious inhibitory effects of siRNAs targeting both *UL18* and *UL19* on different HSV strains, including standard and clinically isolated ACV-resistant viruses. Based on the cocktail therapeutic strategy in HIV-1 treatment, we speculated that the combination of siRNAs targeting *UL18* and *UL19* may enhance the inhibitory effects on these viruses. Therefore, we combined different siRNAs and evaluated the inhibitory effects of these siRNAs using the plaque formation assay. As shown in Figure 5, the combination of siUL18 with siUL19 greatly strengthened the antiviral effects of the siRNAs. The mean proliferation rate of each group was lower than 20%, and some groups even reduced the survival rate to ∼5%. For example, siUL18-3 combined with siUL19-1 reduced the survival rate of HSV-1/F to 2.17%, while the combination of siUL18-1 with siUL19-1 could reduce the survival rate of HSV-1/106 to 2.94%. Taken together, we can draw a conservative conclusion that the interference with siUL18 and siUL19 jointly have a more potent inhibitory effective on the proliferation of different HSV viruses.

### Effect of siRNA Targeting VP5 And VP23 on Virion Maturation

**Figure 6:**
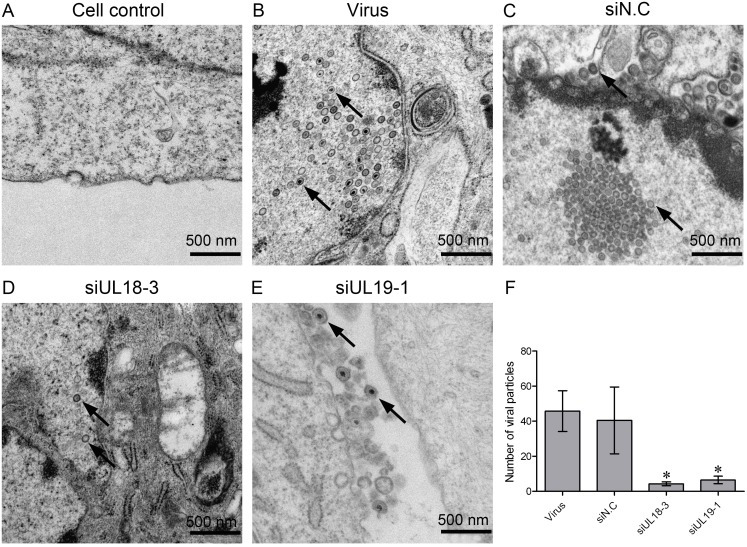
Election microscopy of infected cells. Electron microscopic images of (A) cell control group, (B) viral group, (C) siN.C-treated group, (D) siUL18-3 treated group and (E) siUL19-1 treated group. Black arrows indicate viral particles. Bar, 500 nm. (F) The numbers of particles in each group were counted from six fields and the mean particles per field were calculated. Data are expressed as the mean ± SEM. *P<0.05 vs. viral group.

The capsid is an indispensable component of the HSV virus. From the above results, we found that defects in the expression of VP23 and VP5 greatly inhibited the proliferation of HSV-1, as well as ACV-resistant HSV-1 strains and HSV-2. However, whether the effects were induced by defects in capsid formation or virus egressing from the infected cells was unknown. Thus, to investigate this issue, election microscopy was performed, and virus particles were observed in the nucleus, cytoplasm and extracellular compartments. As shown in figure 6A–E, the viral particles were greatly decreased in siRNA interference groups compared with the virus group and the negative group. The numbers of particles in each group were counted and statistics performed as shown in figure 6F. The inhibition rates on the cellular viral particles by siUL18-3 and siUL19-1 were 91.11% and 86.67%, respectively. Thin sections of HSV-infected cells showed nucleocapsids within the nucleus of both siRNA-treat and untreated Vero cells. The virus group (Figure S1) and negative group (Figure S2) had numerous extracellular virions attached to the cell membrane, as well as a number of virions within the cytoplasm and nucleus. In contrast, after treatment with siUL18 (Figure S3) and siUL19 (Figure S4), numbers of virions at the cell surface and in the nucleus and cytoplasm were significantly decreased.

### VP23 and VP5 Knockdown Decrease the Cellular Viral Genome Load

**Figure 7:**
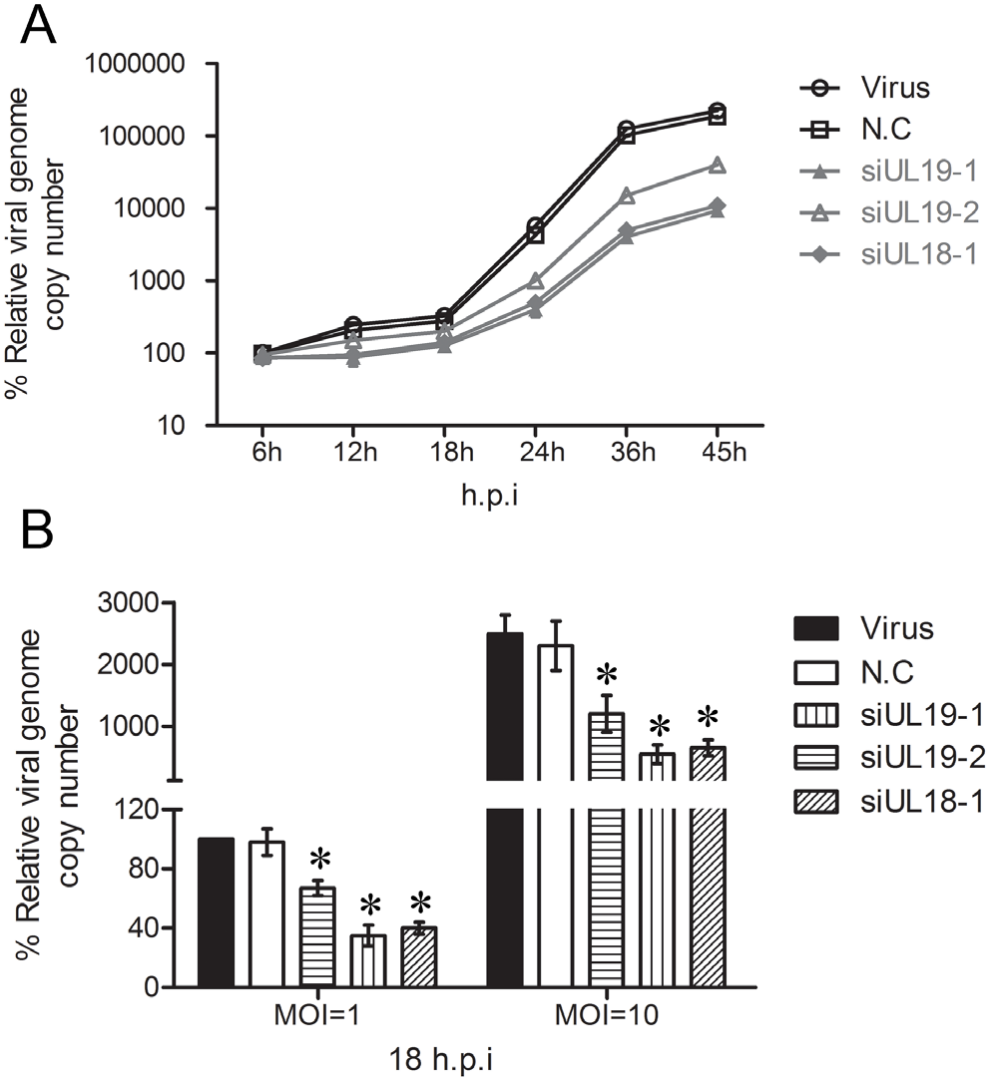
Effects of knockdown VP23 and VP5 on cellular viral genome load. (A) Vero cells were transfected with different siRNAs and infected with HSV-1/F at the multiplicity of infection (MOI) at 1. At the indicated times post-infection, the cellular viral genome DNA was extracted and assayed by quantitative real-time PCR. The results were expressed compared with the genome copies of viral group at 6 hours. (B) The vero cells were transfected with indicated siRNAs and infected with HSV-1/F at different multiplicity of infection (MOI = 1 and MOI = 10). At 18 h post-infection the viral genome of each group was extracted and the genome copy number was quantified. The results were expressed compared with the genome copies of viral group at MOI = 1. Data are expressed as the mean ± SEM. *P<0.05 vs. viral group.

Because it is the viral genome that endows the virus with the ability of pathogenicity, next we test whether siRNA interference with *UL18* and *UL19* can also affect the total viral genome copy numbers in host cells. At different hours after HSV-1 infection, the total intracellular viral genome DNA was extracted and quantified by qRT-PCR. As shown in figure 7A, we can clearly see that compared with the virus control group the intracellular genome copy number of siUL18 and siUL19 interference groups significantly declined. At 45 h post-infection, the genome copy number of the interference group is lower than twentieth compared with the virus control group. The effect of a less efficient siRNA targeting *UL19* (siUL19-2) was also tested, which showed a reduced inhibitory rate when compared with siUL19-1 as expected. Moreover, with different infection virus titers, siRNA decreased the HSV genome copy number clearly and such inhibition efficiency was positively correlated with interference efficiency of siRNA used (compared with siUL19-1 and siUL19-2) (Fig. 7B). From these results, we may safely draw the conclusion that knockdown of VP23 and VP5 can significantly decrease the cellular viral genome load.

## Discussion

Although RNAi is used currently in the development of novel therapeutics and antivirals, only a few studies have explored the potential of this approach for HSV-1. Previous studies have confirmed that siRNAs targeting ICP4, ICP6 and glycoprotein E could inhibit HSV-1 replication *in vitro* [32]–[34]. The capsid is a well-recognized target for antiviral agents due to its crucial role in regulating DNA packaging and maturation. In the present study, we showed that HSV-1 was susceptible to the RNAi pathway. All of the siRNAs tested resulted in the silencing of the HSV-1 capsid gene expression. In several independent experiments, siRNA targeting VP23 and VP5 could greatly affect the proliferation of clinically isolated ACV-resistant HSV-1 strains as well as HSV-1/F and HSV-2/333.

VP5 is a major capsid protein synthesized during the latter phases of HSV-1 gene expression. Interactions have been detected between VP5 and the scaffold protein, triplex protein and VP26, which are important for closure of the capsid shell into an icosahedral structure. Two VP23 molecules combined with one VP19C molecule form a triplex, 320 of which are in each capsid [35]. This triplex structure is important for stabilizing the capsid shell through interactions with adjoining capsomeres consisting of the major capsid protein, VP5 [36]–[37]. Previous studies have shown that HSV-1 strains with mutated forms of VP5 and VP23 failed to form plaques in cell lines [38]. By using a Blast search of coding sequences for VP23 and VP5 in different HSV-1 strains, we found that VP23 and VP5 are highly conserved in different viruses, and even the similarities between HSV-1 and HSV-2 are greater than 87%. These findings suggest that VP5 and VP23 are necessary for HSV-1 to proliferate, opening up possibilities for VP5 and VP23 as new targets in anti-HSV therapies.

The failure of the null mutant in VP19C to form detectable virions has previously been verified by electron microscopy and sedimentation analyses following infection of nonpermissive cells. Cell localization studies also demonstrated the requirement of VP19C for the proper nuclear localization of VP23 [35], [36], [39]. VP26 is not a component of the pre-capsid and not necessary for capsid assembly of HSV-1 [39]–[41]. Thomsen *et al.* [42] and Tatman *et al.* [43] have developed procedures for using recombinant baculoviruses to produce HSV-1 capsids in insect cells. They found that the minimal number of genes required for assembly of capsids is four, including the *UL18*, *UL19* and *UL38* genes (encoding three proteins that form the capsid shell) and either the *UL26.5* or the *UL26* gene. Previous studies have shown that the products of *UL26* and *UL26.5* genes are scaffold proteins, which form a core internal to the capsid shell and interact directly with VP5. These interactions are essential to the assembly of to the icosahedral capsids [44]–[46]. However, in our investigation, silencing *UL26* and *UL26.5* as well as VP19C did not influence HSV-1 replication. Perhaps host cells proteins or other viral proteins can partly compensate for the function of these capsid proteins, but this speculation requires further investigation.

The viral genome is the most important structure of HSV and it endows the virus with the ability of pathogenicity. The process of viral DNA replication and assembly has been well studied. Viral DNA synthesis begins shortly after the appearance of the beta proteins and continues up to 15 h post-infection [47]. Previous studies have shown that there are at least seven HSV-1 genes that are necessary for DNA replication including *UL5*, *UL8*, *UL9*, *UL29*, *UL30*, *UL42*, *UL52* and many host proteins are also involved in this process. These viral proteins involved in DNA replication have provided useful targets for antiviral therapy [48]. After the procapsid was assembled, the viral DNA was packaged in the capsid with the help of several viral proteins such as *UL12* encoding protein. And this process is extremely important for the replication of virus in host cells. It determines whether the virus infection can form a complete infectious progeny virus [12]. In the present study we find that knockdown of capsid protein VP23 and VP5 block the process of capsid formation and disturbance the process of DNA packaging. By quantifying the cellular viral genome copy numbers, we find that blocking the expression of VP23 and VP5 greatly decreased the total viral load in the cells (Fig. 7). The reduction in cellular genome copy numbers after 24 hours should be mainly due to the inhibition of viral secondary infection. Meanwhile, the viral genome copy numbers were also decreased significantly at 18 hours post-infection. One possible reason is that those viral genome DNAs that can’t be packaged were degraded by the nuclease in host cells. However, up to now, degradation mechanisms of the viral genome within the cell were largely unknown. Alternatively, whether these genomes which can’t be packaged may inhibit the replication of the subsequent genome, or the capsid protein VP23 and VP5 may have some unclarified roles in the viral DNA replication requires further experiments to confirm.

HSV causes a variety of human diseases globally, including genital herpes as well as neonatal and sporadic encephalitis. Although a series of antiviral drugs such as ACV and related nucleoside analogs are available for the therapy of HSV infection, it remains highly prevalent worldwide. The resistance to ACV has also created a barrier for the treatment of HSV infections, especially in immunocompromised patients [49]–[50]. Therefore, the development of new, safe and effective anti-HSV agents with different mechanisms of action is a high priority. RNAi has become an advanced tool for screening and identifying gene function, and it may also play an important role in antiviral defense. With inhibition of viral replication successfully demonstrated *in vitro* for a variety of RNA viruses, including rotaviruses, respiratory syncitial virus and Dengue virus, the use of RNAi in the treatment of HSV infections is a strong possibility. Although siRNAs appear to have great promise in antiviral therapy, this technology does have several limitations for clinical applications. Delivery of siRNAs remains the greatest obstacle to their development as therapeutic agents [51]. Direct administration would require siRNAs that are modified to be resistant to nucleases and perhaps conjugated with a ligand for tissue-specific targeting. Additional problems include identification of effective target sites within the target gene, along with potential triggering of interferons and emergence of escape mutants [52]. However, despite these challenges, siRNA technology provides a powerful and promising platform for combating viral infections.

## Conclusions

In conclusion, chemically synthesized siRNAs targeting the HSV-1 capsid protein were demonstrated to silence the expression of HSV-1 capsid proteins efficiently. Interference of expression of capsid proteins VP23 and VP5 could greatly affect the proliferation of clinically isolated ACV-resistant HSV-1 strains as well as HSV-1/F and HSV-2/333. Electron microscopic localization experiments showed that knockdown of VP23 and VP5 greatly impacted the capsid formation of HSV-1. The total cellular viral genome load was also significantly decreased after the knockdown of VP23 and VP5. Thus, our findings suggest that siRNAs targeting VP5 and VP23 can confer excellent antiviral activity by inhibiting HSV-1 replication in cells.

## Supporting Information

Figure S1 Electron microscopic images of the virus control group. Black squares indicate viral particles. Bar, 1 µm.
(TIF)

Figure S2 Electron microscopic images of the negative control group. Black squares indicate viral particles. Bar, 1 µm.
(TIF)

Figure S3 Electron microscopic images of the siUL18-3 treated group. Black squares indicate viral particles. Bar, 2 µm.
(TIF)

Figure S4 Electron microscopic images of the siUL19-1 treated group. Black squares indicate viral particles. Bar, 2 µm.
(TIF)
