# Supplementary figures and images for: Silencing Herpes Simplex Virus Type 1 Capsid Protein Encoding Genes by siRNA: A Promising Antiviral Therapeutic Approach
Source: PLoS One. 2014 May 2;9(5):e96623. doi: 10.1371/journal.pone.0096623 (PMC4008601; doi:10.1371/journal.pone.0096623)

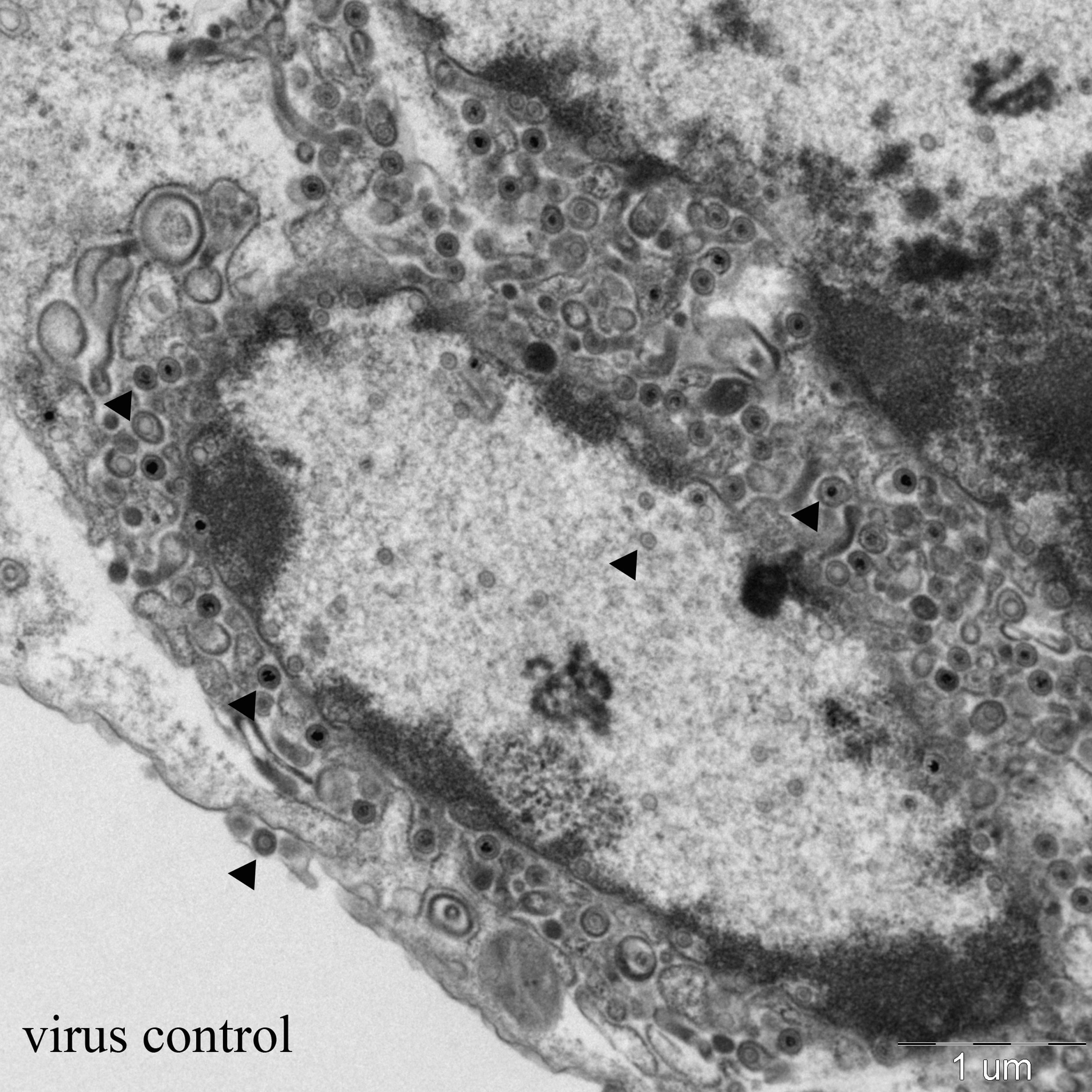

Supplement: Figure S1 — Electron microscopic images of the virus control group. Black squares indicate viral particles. Bar, 1 µm. (TIF) [file pone.0096623.s001.tif]

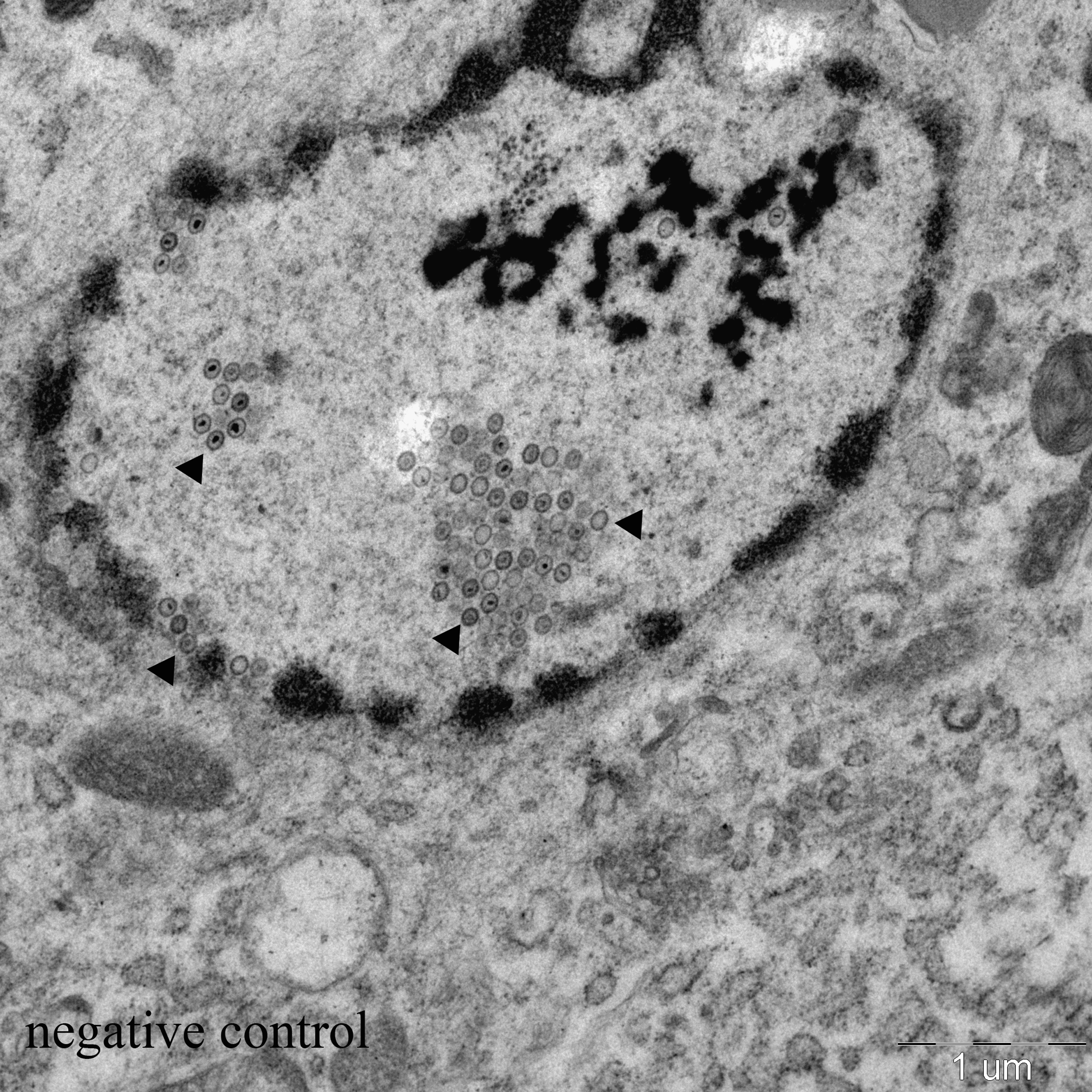

Supplement: Figure S2 — Electron microscopic images of the negative control group. Black squares indicate viral particles. Bar, 1 µm. (TIF) [file pone.0096623.s002.tif]

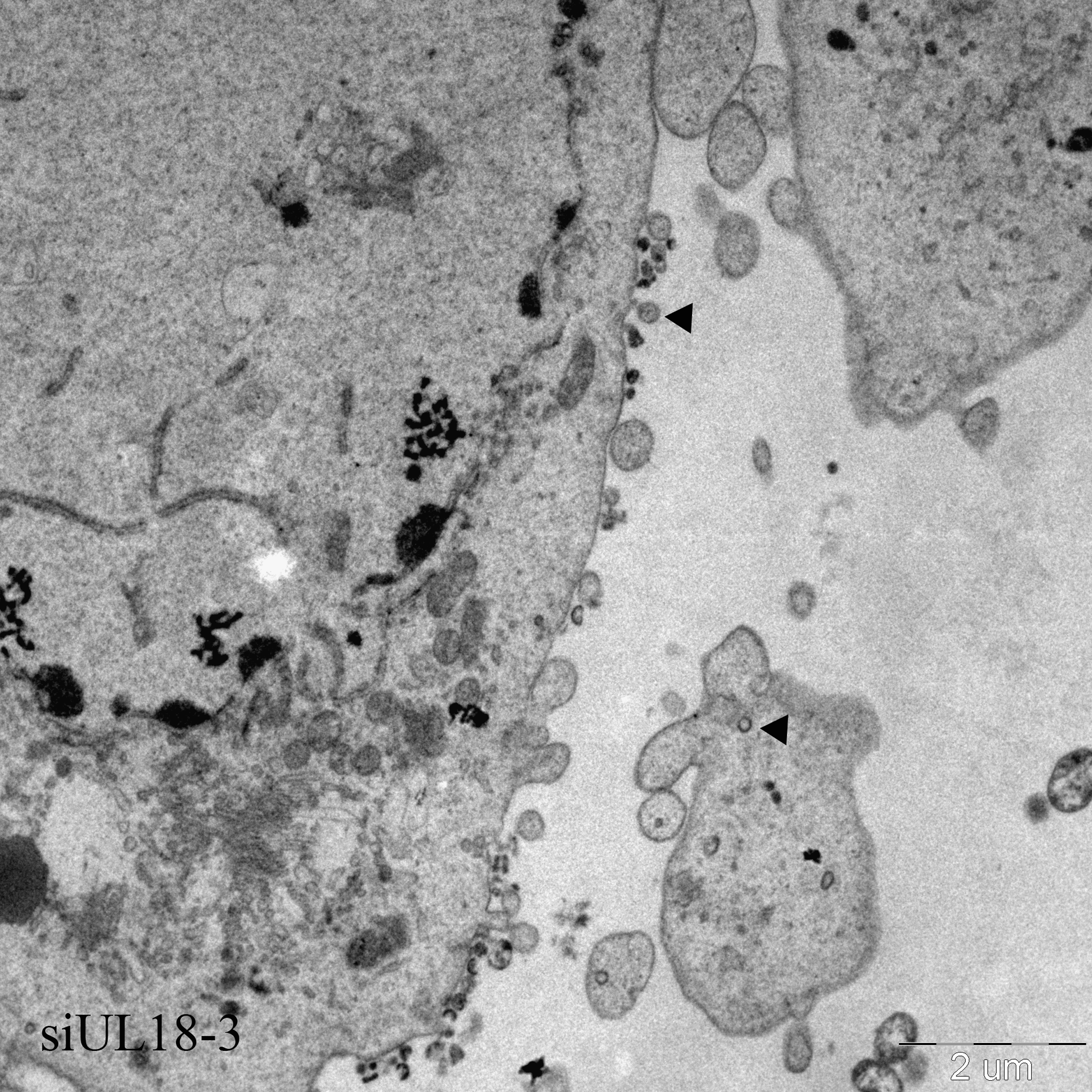

Supplement: Figure S3 — Electron microscopic images of the siUL18-3 treated group. Black squares indicate viral particles. Bar, 2 µm. (TIF) [file pone.0096623.s003.tif]

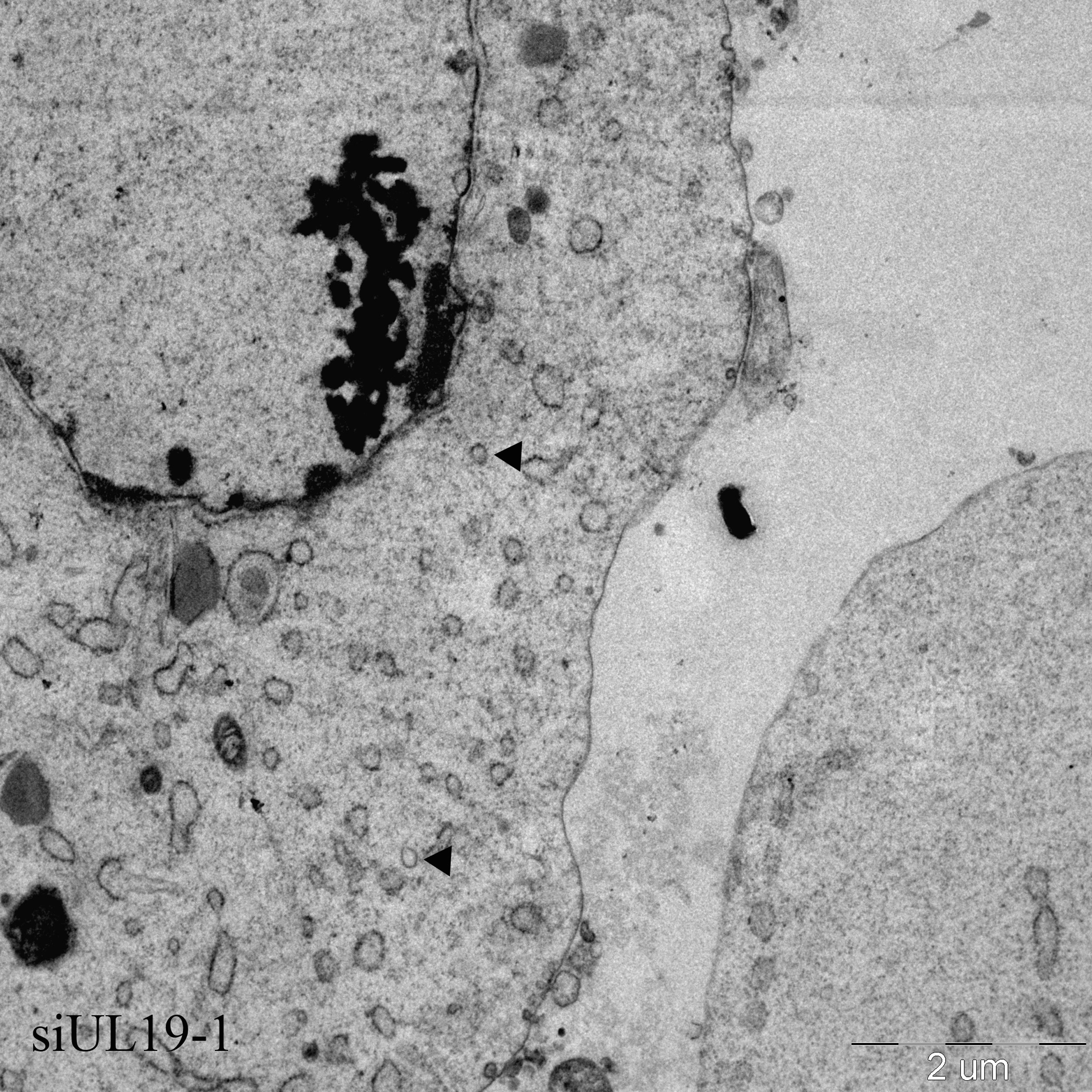

Supplement: Figure S4 — Electron microscopic images of the siUL19-1 treated group. Black squares indicate viral particles. Bar, 2 µm. (TIF) [file pone.0096623.s004.tif]
